# Cognitive Targeted Prostate Biopsy Alone for Diagnosing Clinically Significant Prostate Cancer in Selected Biopsy-Naïve Patients: Results from a Retrospective Pilot Study

**DOI:** 10.3390/diagnostics14151643

**Published:** 2024-07-30

**Authors:** Michelangelo Olivetta, Celeste Manfredi, Lorenzo Spirito, Carmelo Quattrone, Francesco Bottone, Marco Stizzo, Ugo Amicuzi, Arturo Lecce, Andrea Rubinacci, Lorenzo Romano, Giampiero Della Rosa, Salvatore Papi, Simone Tammaro, Paola Coppola, Davide Arcaniolo, Ferdinando Fusco, Marco De Sio

**Affiliations:** 1 Department of Urology, AOU San Giovanni e Ruggi D’Aragona, G. Fucito Hospital, 84085 Mercato San Severino, Italy; 2 Unit of Urology, Department of Woman, Child and General and Specialized Surgery, University of Campania “Luigi Vanvitelli”, 80131 Naples, Italy; 3 Division of Urology, Department of Surgical Sciences, AORN Sant’Anna e San Sebastiano, 81100 Caserta, Italy

**Keywords:** clinically significant prostate cancer, transrectal prostate biopsy, cognitive targeted prostate biopsy

## Abstract

(1) Background: To identify a particular setting of biopsy-naïve patients in which it would be reasonable to offer only cognitive targeted prostate biopsy (PBx) with a transrectal approach. (2) Methods: We designed an observational retrospective pilot study. Patients with a prostatic specific antigen (PSA) level > 10 ng/mL, either a normal or suspicious digital rectal examination (DRE), and a lesion with a PI-RADS score ≥ 4 in the postero-medial or postero-lateral peripheral zone were included. All patients underwent a transrectal PBx, including both systematic and targeted samples. The detection rate of clinically significant prostate cancer (csPCa) (Gleason Score ≥ 7) was chosen as the primary outcome. We described the detection rate of csPCa in systematic PBx, targeted PBx, and overall PBx. (3) A total of 92 patients were included. Prostate cancer was detected in 84 patients (91.30%) with combined biopsies. A csPCa was diagnosed in all positive cases (100%) with combined biopsies. Systematic PBxs were positive in 80 patients (86.96%), while targeted PBxs were positive in 84 men (91.30%). Targeted PBx alone would have allowed the diagnosis of csPCa in all positive cases; systematic PBx alone would have missed the diagnosis of 8/84 (9.52%) csPCa cases (4 negative patients and 4 not csPCa) (*p* = 0.011). (4) Conclusions: Cognitive targeted PBx with a transrectal approach could be offered alone to diagnose csPCa in biopsy-naïve patients with PSA ≥ 10 ng/mL, either normal or suspicious DRE, and a lesion with PI-RADS score ≥ 4 in the postero-medial or postero-lateral peripheral zone.

## 1. Introduction

Prostate biopsy (PBx) is currently the gold standard for the diagnosis of prostate cancer (PCa). PCa is usually suspected on the basis of digital rectal examination (DRE) and/or prostatic specific antigen (PSA) levels, but the definitive diagnosis depends on histopathological detection of adenocarcinoma in PBx cores. In clinical practice, according to European Association of Urology (EAU) recommendations, a multiparametric magnetic resonance imaging (mpMRI) of the prostate should be performed before the PBx [1]. mpMRI is the tool used to evaluate the probability of a clinically significant prostate cancer (csPCa) according to the Prostate Imaging–Reporting and Data System (PI-RADS) score. For PI-RADS v2.1, csPCa is pathologically defined as a Gleason score ≥ 7, and/or a volume > 0.5 cc, and/or extraprostatic extension (EPE) [2]. PI-RADS 4–5 are associated with csPCa in approximately 62% of cases [3]. In this regard, it is important to underline that in recent years there has been much debate on the need for diagnosis and treatment of GS 6 tumors, with some authors even questioning the definition of tumor for GS 6 lesions [4].

In common clinical practice, according to EAU recommendations, combined systematic (sPBx) and targeted (tPBx) biopsies are generally performed when PCa is suspected and mpMRI is positive (PI-RADS ≥ 3); although this choice is widely accepted for biopsy-naïve patients, tPBx alone could be offered to non-biopsy-naïve subjects with positive mpMRI (PI-RADS ≥ 3) [1]. tPBx appears to be associated with greater detection of GS ≥ 7 tumors but less identification of GS 6 cancers than sPBx [5]. It can be performed with different image-guided techniques (cognitive, fusion, and direct-in-bore); however, a clear superiority of one over the others has not been demonstrated [6]. Therefore, the choice is generally based on the urologist’s preference, with the cognitive technique probably remaining the most used, for costs and simplicity of execution [7,8]. There is no sufficient evidence to recommend tPBx alone in biopsy-naïve patients, but it is reasonable to hypothesize that there are some characteristics that may guide towards this specific diagnostic choice in selected subjects.

Finally, PBx can be performed with a transrectal or transperineal approach; there is no clear superiority of one technique over the other in terms of cancer detection rate, but the transperineal technique would be preferable due to the lower risk of infections related to the procedure [9]. However, transrectal access still seems to be the most practiced and could have some advantages for posterior lesions, which could be better sampled [10].

The aim of this study was to identify a particular setting of biopsy-naïve patients in which it would be reasonable to offer only cognitive tPBx with a transrectal approach.

## 2. Materials and Methods

### 2.1. Study Design and Ethical Details

We designed an observational retrospective pilot study involving consecutive patients referring to our center (Unit of Urology, Department of Woman, Child and General and specialized Surgery, University of Campania “Luigi Vanvitelli”, Naples, Italy) from May 2021 to September 2023 for suspected PCa. All patients gave written informed consent for the collection and publication of their clinical data. Due to the purely observational, retrospective nature of the study, after consultation with the Ethics Committee, it was exempted from specific approval.

### 2.2. Patient Enrollment and Measured Variables 

Male patients, ≥18 years old, with PSA level > 10 ng/mL, either normal or suspicious DRE, and a single lesion with PI-RADS score ≥ 4 in the postero-medial or postero-lateral peripheral zone, undergoing PBx were included in the study. Previous PBx and prostate surgery were considered exclusion criteria. Subjects with a history of recurrent or chronic prostatitis, indwelling bladder catheter, pelvic radiotherapy, and anorectal surgery were also excluded. Patients with concomitant lesions with a PI-RADS score 3 in the peripheral zone, multiple lesions with a PI-RADS score ≥ 4 in the peripheral zone, or lesions with a PI-RADS score ≥ 3 in the transitional zone were not included.

All subjects were evaluated by experienced urologists from our center. PSA tests and mpMRI were performed in different centers depending on the patient’s choice. Age, ethnicity, body mass index (BMI), family history of cancer, PSA, DRE, PI-RADS score, length of lesion, prostate volume (according to mpMRI), drugs, and medical history were collected for each patient. All biopsy specimens were examined by expert pathologists from our center. The characteristics of the biopsy specimens (number, length, presence of PCa, and GS) were recorded. Intraoperative and postoperative complications were described. Postoperative adverse events were classified according to the Clavien–Dindo (CD) system.

The detection rate of csPCa was chosen as the primary outcome. It was defined as a lesion with a GS ≥ 7, regardless of tumor volume and extension. We described the detection rate of csPCa in sPBx, tPBx, and overall PBx (sPBx + tPBx). Intraoperative and post-biopsy complications were selected as secondary outcomes.

### 2.3. Biopsy Technique 

**Figure 1:**
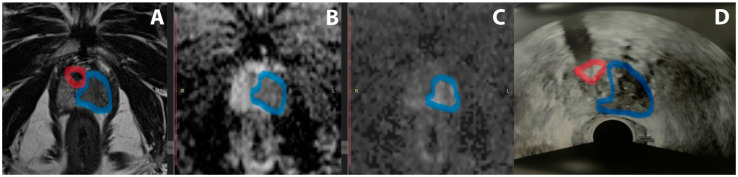
Example of PI-RADS 5 lesion. T2 (**A**), ADC (**B**), DWI (**C**), and mpMRI sequences and TRUS (**D**). The suspicious lesion is highlighted in blue, and a calcification used as a reference point for performing the tPBx is highlighted in red. mpMRI: multiparametric magnetic resonance imaging; PI-RADS: Prostate Imaging–Reporting and Data System; tPBx: targeted prostate biopsy; TRUS: transrectal ultrasound.

All patients underwent a transrectal PBx, including both systematic and targeted samples. sPBx was performed in the peripheral zone, including 12 samples (6 per lobe). tPBx was performed with the cognitive technique (example in Figure 1), including at least 2 samples of a PI-RADS score ≥ 4 lesion in the peripheral zone. A BK3000 (BK Medical, Burlington, MA, USA) was used as the ultrasound machine. BARD disposable biopsy guns (18 g × 20 cm, 22 mm) were used for biopsy sampling (BARD Medical, Covington, GA, USA). Fosfomycin 3 g was administered orally 1–3 h before the procedure as antibiotic prophylaxis, following local antimicrobial use policy. PBx was preceded by disinfection of the rectal ampulla with povidone–iodine and local anesthesia with mepivacaine. After PBx, the patients were observed for approximately 2 h before discharge. All procedures were performed by experienced urologists in the outpatient clinic.

### 2.4. Statistics

The categorical variables were described as frequencies and percentages. The continuous variables were expressed as medians and interquartile ranges (IQRs). The Shapiro–Wilk test was applied as a normality test. The McNemar test was used to compare the detection rate of csPCa in tPBx and sPBx. Statistical significance was set a priori at *p* ≤ 0.05. The IBM Statistical Package for the Social Sciences (IBM Corp. Released 2015. IBM SPSS Statistics for Windows, Version 23.0. Armonk, NY, USA) was used for the statistical analyses.

## 3. Results

A total of 92 patients were included in the study. The median (IQR) age was 68 (63–68.5) years, the median (IQR) PSA was 15.5 (11.3–55.8) ng/mL, and a suspicious DRE was recorded in 76 (82.61%) cases. PI-RADS 4 and 5 lesions were identified in 48 (52.17%) and 44 (47.83%) subjects, respectively. The median (IQR) maximum length of PI-RADS ≥ 4 lesions on mpMRI was 14 (12–22) mm. The baseline characteristics of the patients are summarized in Table 1.

PCa was detected in 84 patients (91.30%). In other words, the overall PCa detection rate using either sPBx or tPBx was 91.30%. A csPCa was diagnosed in all positive cases (100%) with combined biopsies. sPBxs were positive in 80 patients (86.96%), while tPBxs were positive in 84 men (91.30%). In four cases (4.34%), tPBx alone tested positive, while sPBx alone never tested positive. tPBx alone would have allowed the diagnosis of csPCa in all positive cases; on the contrary, sPBx alone would have missed the diagnosis of 8/84 (9.52%) csPCa (4 negative patients and 4 not csPCa), in a statistically significant way (*p* = 0.011). The histological characteristics are detailed in Table 2. 

No intraoperative complication occurred. No local or systemic infection was recorded after biopsy. Only 2 (2.17%) cases of acute urinary retention (CD Grade I) were described.

## 4. Discussion

Several methods have been proposed over the years to improve the detection of PCa, but biopsy remains the cornerstone for definitive diagnosis [11,12,13,14,15,16,17]. To date, only for intermediate-risk csPCa low-volume is a sPBx mandatory. According to EAU guidelines, csPCa with low-volume ISUP grade 2 (PSA < 10 ng/mL, low density, clinical stage < cT2a, and a low number of positive systematic cores) can be considered for active surveillance and therefore the burden of the disease should be evaluated [18]. Excluding this subgroup, our results show that, in a strictly selected court of patients, sPBx could be omitted, as no differences were recorded in the detection rate compared to tPBx alone, reducing the time, the invasiveness of the procedure, and possible adverse effects. 

These data seem to contrast with the current literature. Drost et al. performed a Cochrane pooled analysis and found that for every 100 biopsy-naïve men with a positive MRI, MRI used with tPBx detected approximately 39 men with csPCa, and sPBx detected 5 additional cases [5]. In the MRI-FIRST multicenter trial, it is stated that tumor detection was improved when sPBx and tPBx were combined [19] and also the Precision trial suggests that the use of tPBx alone could decrease the detection of non-significant prostate cancer, but, on the other side, adding sPBx could still be helpful for the detection of ISUP grade group 2 or higher cancers, which might be optimized when both approaches are combined. [20]. It should be taken into consideration that all the previous studies considered a heterogeneous, non-selected population, and the biopsies were performed with different biopsy techniques. On the contrary, we selected a specific population of patients with precise characteristics in terms of PI-RADS lesions and location on the whole gland, and we offered standardized procedure, taking advantage of its main features. Ahdoot et al. included in their analysis only patients with a PI-RADS score of 5 and showed that targeted biopsies alone could be used, with a low risk of missing csPC (1%) [21]. In patients with PI-RADS 5 on mpMRI and a PSA density > 0.15 ng/mL^2^, the role of systematic biopsy is negligible and can be omitted in this population [22].

The detection of a population of selected patients who can benefit from performing tPBx alone could have important positive implications. In fact, complications related to the PBx procedure, in addition to the type of procedure used itself, are directly proportional to the number of samples taken. [23]

With this study, we have highlighted a concrete setting of biopsy-naïve patients in which tPBx alone could be proposed. Fewer biopsies needed could be associated with fewer complications and shorter times both for performing the biopsy and for histological interpretation of the samples, with consequent clinical and economic advantages. Obviously, our encouraging results should be confirmed with large-sample randomized studies, and the supposed clinical and economic advantages demonstrated with ad hoc research. 

The choice of the prostate biopsy technique deserves a separate discussion. Transrectal PBx seems to have a significantly higher risk of infections than transperineal PBx because the needle passes through the rectum, allowing bacteria to migrate to the prostate, urinary system, or bloodstream. For this reason, current European guidelines recommend transrectal PBx as a second choice; however, it remains the most commonly performed procedure of the two [1]. At the same time, American guidelines still recommend both approaches due to insufficient evidence on the infection risk to favor one over the other [24]. Overall, there is no clear evidence of the superiority of one approach over the other in terms of PCa detection rate. The transrectal technique offers easier access to certain prostate zones, such as the base and posterior surface, but poses challenges for other zones, such as the anterior apex region, due to the depth required and the risk of urethral penetration [25,26].

This study should be read and interpretated taking into account several limitations. The small sample size, the retrospective design, and the absence of a control group are the main weaknesses of our research. Furthermore, several techniques described (mpMRI, PBx, and pathological examination) are highly operator-dependent and influenced by the single-center nature of our study. Finally, the characteristics of the biopsy-naïve patients were chosen arbitrarily; therefore, there is no certainty that they are the best possible for our purposes.

## 5. Conclusions

According to our preliminary experience, in biopsy-naïve patients with PSA ≥ 10 ng/mL, either a normal or suspicious DRE, and a lesion with PI-RADS score ≥ 4 in the postero-medial or postero-lateral peripheral zone, cognitive tPBx with a transrectal approach could be offered alone to diagnose csPCa. Further large, randomized studies should confirm our promising findings.

## Figures and Tables

**Table 1:** Baseline characteristics of patients (n = 92).

**Age,** Median (IQR)	
*years*	68 (63–68.5)
**Ethnicity**, n (%)	
Caucasian	92 (100)
**BMI**, Median (IQR)	
*points*	24.9 (23.5–28)
**Prostate volume**, Median (IQR)	
*mL*	47 (34–54)
**Family history for PCa or BCa**, n (%)	10 (10.87)
**PSA**, Median (IQR)	
*ng/mL*	15.5 (11.3–55.8)
**Suspicious DRE**, n (%)	76 (82.60)
**PI-RADS score**, n (%)	
4	48 (52.17)
5	44 (47.83)
**Max length of lesion**, Median (IQR)	
*mm*	14 (12–22)
**Anticoagulant or antiplatelet agents**, n (%)	46 (50.0)
**BPH medications**, n (%)	44 (47.83)

BCa: breast cancer; BMI: body mass index; BPH: Benign Prostatic Hyperplasia; DRE: digital rectal examination; IQR: interquartile range; PCa: prostate cancer; PI-RADS: Prostate Imaging–Reporting and Data System; PSA: Prostate-Specific Antigen.

**Table 2:** Histological characteristics of prostate biopsy specimens.

**Systematic biopsies per patient**, Median (IQR)	12 (12–12)
**Targeted biopsies per patient**, Median (IQR)	3 (2–3)
**Total length of specimens**, Median (IQR) *mm*	
Systematic	138.5 (119.5–145.75)
Targeted	35 (27.0–43.75)
Total	170.5 (153.25–184.5)
**Presence of PCa**, n (%)	
Yes	84 (91.30)
No	8 (8.70)
**Overall GS**, n (%)	
6	0 (0)
7 (3 + 4)	16 (19.05)
7 (4 + 3)	16 (19.05)
8–10	52 (61.90)
≥7	84 (100)
**Diagnosis of PCa according to type of PBx**, n (%)	
Systematic	80 (86.96)
Targeted	84 (91.30)
Systematic + Targeted	84 (91.30)
**Diagnosis of csPCa according to type of PBx**, n (%)	
Systematic	76 (90.48)
Targeted	84 (100)
Systematic + Targeted	84 (100)

csPCa: clinically significant prostate cancer; GS: Gleason score; IQR: interquartile range; PBx: prostate biopsy; PCa: prostate cancer.

## Data Availability

Raw data available upon appropriate request to the corresponding author.

## References

[1] Cornford P., van den Bergh R.C., Briers E., Broeck T.V.D., Brunckhorst O., Darraugh J., Eberli D., De Meerleer G., De Santis M., Farolfi A. (2024). EAU-EANM-ESTRO-ESUR-ISUP-SIOG Guidelines on Prostate Cancer-2024 Update. Part I: Screening, Diagnosis, and Local Treatment with Curative Intent. Eur. Urol..

[2] Turkbey B., Rosenkrantz A.B., Haider M.A., Padhani A.R., Villeirs G., Macura K.J., Tempany C.M., Choyke P.L., Cornud F., Margolis D.J. (2019). Prostate Imaging Reporting and Data System Version 2.1: 2019 Update of Prostate Imaging Reporting and Data System Version 2. Eur. Urol..

[3] Schoots I.G., Padhani A.R. (2021). Risk-adapted biopsy decision based on prostate magnetic resonance imaging and prostate-specific antigen density for enhanced biopsy avoidance in first prostate cancer diagnostic evaluation. BJU Int..

[4] Shill D.K., Roobol M.J., Ehdaie B., Vickers A.J., Carlsson S.V. (2021). Active surveillance for prostate cancer. Transl. Androl. Urol..

[5] Drost F.-J.H., Osses D.F., Nieboer D., Steyerberg E.W., Bangma C.H., Roobol M.J., Schoots I.G. (2019). Prostate MRI, with or without MRI-targeted biopsy, and systematic biopsy for detecting prostate cancer. Cochrane Database Syst. Rev..

[6] Wegelin O., van Melick H.H., Hooft L., Bosch J.R., Reitsma H.B., Barentsz J.O., Somford D.M. (2017). Comparing Three Different Techniques for Magnetic Resonance Imaging-targeted Prostate Biopsies: A Systematic Review of In-bore versus Magnetic Resonance Imaging-transrectal Ultrasound fusion versus Cognitive Registration. Is There a Preferred Technique?. Eur. Urol..

[7] Chang S.D., Ghai S., Kim C.K., Oto A., Giganti F., Moore C.M. (2021). MRI Targeted Prostate Biopsy Techniques: AJR Expert Panel Narrative Review. AJR Am. J. Roentgenol..

[8] Altok M., Kim B., Patel B.B., Shih Y.-C.T., Ward J.F., McRae S.E., Chapin B.F., Pisters L.L., Pettaway C.A., Kim J. (2018). Cost and efficacy comparison of five prostate biopsy modalities: A platform for integrating cost into novel-platform comparative research. Prostate Cancer Prostatic Dis..

[9] Xiang J., Yan H., Li J., Wang X., Chen H., Zheng X. (2019). Transperineal versus transrectal prostate biopsy in the diagnosis of prostate cancer: A systematic review and meta-analysis. World J. Surg. Oncol..

[10] Kaneko M., Medina L.G., Lenon M.S.L., Hemal S., Sayegh A.S., Jadvar D.S., Ramacciotti L.S., Paralkar D., Cacciamani G.E., Lebastchi A.H. (2023). Transperineal vs transrectal magnetic resonance and ultrasound image fusion prostate biopsy: A pair-matched comparison. Sci. Rep..

[11] Ditonno F., Franco A., Manfredi C., Veccia A., Valerio M., Bukavina L., Zukowski L.B., Vourganti S., Stenzl A., Andriole G.L. (2024). Novel non-MRI imaging techniques for primary diagnosis of prostate cancer: Micro-ultrasound, contrast-enhanced ultrasound, elastography, multiparametric ultrasound, and PSMA PET/CT. Prostate Cancer Prostatic Dis..

[12] Fusco F., Emberton M., Arcaniolo D., De Nunzio C., Manfredi C., Creta M. (2023). Prostatic high-resolution micro-ultrasound: An attractive step-forward in the management of prostate cancer patients. Prostate Cancer Prostatic Dis..

[13] Manfredi C., Fernández- Pascual E., Linares-Espinós E., Couñago F., Martínez-Salamanca J.I. (2021). New Frontiers In Focal Therapy For Prostate Cancer: PSMA PET/MRI. World J. Clin. Oncol..

[14] Manfredi C., Fernández-Pascual E., Arcaniolo D., Emberton M., Sanchez-Salas R., Guix C.A., Bianco F., Cathcart P., Murphy D.G., Couñago F. (2022). The Role of Prostate-specific Membrane Antigen Positron Emission Tomography/Magnetic Resonance Imaging in Primary and Recurrent Prostate Cancer: A Systematic Review of the Literature. Eur. Urol. Focus..

[15] Bologna E., Ditonno F., Licari L.C., Franco A., Manfredi C., Mossack S., Pandolfo S.D., De Nunzio C., Simone G., Leonardo C. (2024). Tissue-Based Genomic Testing in Prostate Cancer: 10-Year Analysis of National Trends on the Use of Prolaris, Decipher, ProMark, and Oncotype DX. Clin. Pract..

[16] Fernández-Pascual E., Manfredi C., Martín C., Martínez-Ballesteros C., Balmori C., Lledó-García E., Quintana L.M., Curvo R., Carballido-Rodríguez J., Bianco F.J. (2022). mpMRI-US Fusion-Guided Targeted Cryotherapy in Patients with Primary Localized Prostate Cancer: A Prospective Analysis of Oncological and Functional Outcomes. Cancers.

[17] Bologna E., Licari L.C., Franco A., Ditonno F., Manfredi C., De Nunzio C., Antonelli A., De Sio M., Leonardo C., Simone G. (2024). Incidental Prostate Cancer in Patients Treated for Benign Prostatic Hyperplasia: Analysis from a Contemporary National Dataset. Diagnostics.

[18] Lam T.B., MacLennan S., Willemse P.-P.M., Mason M.D., Plass K., Shepherd R., Baanders R., Bangma C.H., Bjartell A., Bossi A. (2019). EAU-EANM-ESTRO-ESUR-SIOG Prostate Cancer Guideline Panel Consensus Statements for Deferred Treatment with Curative Intent for Localised Prostate Cancer from an International Collaborative Study (DETECTIVE Study). Eur. Urol..

[19] Rouvière O., Puech P., Renard-Penna R., Claudon M., Roy C., Mège-Lechevallier F., Decaussin-Petrucci M., Dubreuil-Chambardel M., Magaud L., Remontet L. (2019). Use of prostate systematic and targeted biopsy on the basis of multiparametric MRI in biopsy-naive patients (MRI-FIRST): A prospective, multicentre, paired diagnostic study. Lancet Oncol..

[20] Luzzago S., de Cobelli O., Mistretta F.A., Piccinelli M.L., Lorusso V., Morelli M., Bianchi R., Catellani M., Cozzi G., Di Trapani E. (2021). MRI-targeted or systematic random biopsies for prostate cancer diagnosis in biopsy naïve patients: Follow-up of a PRECISION trial-like retrospective cohort. Prostate Cancer Prostatic Dis..

[21] Ahdoot M., Lebastchi A.H., Long L., Wilbur A.R., Gomella P.T., Mehralivand S., Daneshvar M.A., Yerram N.K., O’connor L.P., Wang A.Z. (2022). Using Prostate Imaging-Reporting and Data System (PI-RADS) Scores to Select an Optimal Prostate Biopsy Method: A Secondary Analysis of the Trio Study. Eur. Urol. Oncol..

[22] Tafuri A., Iwata A., Shakir A., Iwata T., Gupta C., Sali A., Sugano D., Mahdi A.S., Cacciamani G.E., Kaneko M. (2021). Systematic Biopsy of the Prostate can Be Omitted in Men with PI-RADS™ 5 and Prostate Specific Antigen Density Greater than 15. J. Urol..

[23] Wegelin O., Exterkate L., van der Leest M., Kelder J.C., Bosch J.R., Barentsz J.O., Somford D.M., van Melick H.H. (2019). Complications and Adverse Events of Three Magnetic Resonance Imaging-based Target Biopsy Techniques in the Diagnosis of Prostate Cancer Among Men with Prior Negative Biopsies: Results from the FUTURE Trial, a Multicentre Randomised Controlled Trial. Eur. Urol. Oncol..

[24] Wei J.T., Barocas D., Carlsson S., Coakley F., Eggener S., Etzioni R., Fine S.W., Han M., Kim S.K., Kirkby E. (2023). Early detection of prostate cancer: AUA/SUO guideline part II: Considerations for a prostate biopsy. J. Urol..

[25] Dell’Atti L., Slyusar V., Ronchi P., Manno S., Cambise C. (2024). Transrectal Prostate Biopsy Approach in Men Undergoing Kidney Transplant: A Retrospective Cohort Study at Three Referral Academic Centers. Diagnostics.

[26] Cauni V.M., Stanescu D., Tanase F., Mihai B., Persu C. (2021). Magnetic resonance/ultrasound fusion targeted biopsy of the prostate can be improved by adding systematic biopsy. Med. Ultrason..

